# On the uncertainty beneath the name *Oithona similis* Claus, 1866 (Copepoda, Cyclopoida)

**DOI:** 10.3897/zookeys.552.6083

**Published:** 2016-01-13

**Authors:** Georgina D. Cepeda, Marina E. Sabatini, Cristina L. Scioscia, Fernando C. Ramírez, María D. Viñas

**Affiliations:** 1 Instituto de Investigaciones Marinas y Costeras (IIMyC), Facultad de Ciencias Exactas y Naturales, Universidad Nacional de Mar del Plata, Consejo Nacional de Investigaciones Científicas y Técnicas (CONICET), Funes 3350, B7602AYL Mar del Plata, Argentina; 2 Instituto Nacional de Investigación y Desarrollo Pesquero (INIDEP), Paseo Victoria Ocampo No 1, B7602HSA Mar del Plata, Argentina; 3 Museo Argentino de Ciencias Naturales Bernardino Rivadavia (CONICET-MACNBR), Avenida Ángel Gallardo 470, C1405DJR Buenos Aires, Argentina

**Keywords:** Nomenclature, Oithona helgolandica Claus, 1863, Oithona similis Claus, 1866, sequence databases, taxonomy

## Abstract

The marine cyclopoid *Oithona similis* *sensu lato* Claus, 1866, is considered to be one of the most abundant and ubiquitous copepods in the world. However, its minimal original diagnosis and the unclear connection with its (subjective) senior synonym *Oithona helgolandica* Claus, 1863, may have caused frequent misidentification of the species. Consequently, it seems possible that several closely related but distinct forms are being named *Oithona similis* or *Oithona helgolandica* without explicit and accurate discrimination. Here the current situation concerning the correct assignment of the two species is revised, the morphological characters commonly used to identify and distinguish each species are summarized, and the nomenclatural implications of indiscriminately using these names in current taxonomic and ecological practice is considered. It is not intended to upset a long-accepted name in its accustomed meaning but certainly the opposite. “In pursuit of the maximum stability compatible with taxonomic freedom” (International Commission of Zoological Nomenclature), we consider that reassessment of the diagnostic characters of *Oithona similis* *sensu stricto* cannot be postponed much longer. While a consensus on taxonomy and nomenclatural matters can be attained, we strongly recommend specifically reporting the authority upon which the identification of either *Oithona similis* s.l. or *Oithona helgolandica* s.l. has been accomplished.

## Introduction

A global-scale baseline assessment of marine zooplankton biodiversity is critically needed to provide a contemporary benchmark against which future environmental changes can be evaluated (Bucklin et al. 2011). The largest obstacle for most zooplankton taxa is the difficulty in identifying specimens, which has resulted in marked under-specification of species and morphological types. The small cyclopoid *Oithona similis* Claus, 1866 is recognized as one of the most important marine copepods in terms of both abundance and breadth of distribution, occupying a key position in the global oceans (Galliene and Robins 2001). However, there is still much confusion regarding not only characters for its recognition but also its name.

*Oithona similis* was first described by Claus in 1866 from specimens collected in the Mediterranean Sea, near Nice, France. Three years earlier, the same author had described a very similar congener from waters off Helgoland (North Sea) that he named *Oithona helgolandica* (Claus 1863). The original description of the two species were too brief, unfortunately, to allow for adequate discrimination of the two species, and the synonymy between them has been under discussion ever since.

In our opinion, a rather confusing subjective synonymy of the two names has developed in recent practice, and the junior name *similis* has been imposed over *helgolandica* by prevailing usage, which is in clear contravention of the Principle of Priority (International Commission on Zoological Nomenclature, hereafter ICZN 1999, Article 23). Both specific names have coexisted since 1866, and both are currently in use depending upon authors’ taxonomic judgment. In our own experience, the replacement of the name *Oithona helgolandica* by *Oithona similis* has very often been requested by reviewers located worldwide, even when there is the possibility that the two names may refer to two distinctive taxa.

Among contemporary records, references to *Oithona similis* are plentiful from almost everywhere in the world’s oceans (Razouls et al. 2005–2015), while references to *Oithona helgolandica* appear to be restricted to relatively few regions: NW and N Iberian shelf (e.g., Cabal et al. 2008), Gulf of Lion (e.g., Razouls 1972), Ligurean Sea (e.g., Pane et al. 2005), Tyrrhenian and Ionian seas (e.g., Vaissiere and Seguin 1980), SW Mediterranean Sea and Atlantic coast of Morocco (e.g., Hafferssas and Seridji 2010) and Red Sea (e.g., Vaissiere and Seguin 1980). In the SW Atlantic both names have been recorded: *Oithona similis* has been used by, for example, Pallares (1968), Björnberg (1981), Mazzochi et al. (1995), Viñas et al. (2002), Fernández-Severini and Hoffmeyer (2005), Aguirre et al. (2012), Cepeda et al. (2012), Thompson et al. (2012), and *Oithona helgolandica* by, for example, Ramírez (1970), Sabatini and Martos (2002), Viñas et al. (2013), Antacli et al. (2014), and Temperoni et al. (2014).

New approaches such as molecular tools are becoming increasingly attractive for identifying plankton. Advancements, however, depend largely on the provision of reference libraries with sequences coming from accurately identified individuals (Lindeque et al. 2013). There is the urgent need to clarify these issues, so both morphological and metagenetic global databases can be refined before upcoming studies enhance the current confusion. Rather than trying to prove a point, we review here the historical debate going back to the assignment of the two specific names, summarize the arguments that give support to the hypothesis that *Oithona similis* and *Oithona helgolandica* are not truly objective synonyms and discuss the implications of using both names in present times without exacting discrimination. “In pursuit of the maximum stability compatible with taxonomic freedom” (ICZN 1999; Principle #4), we consider that reassessment of the diagnostic characters of *Oithona similis* s.s. and *Oithona helgolandica* s.s. cannot be postponed.

## Historical background

Original diagnoses of *Oithona helgolandica* and *Oithona similis* were in both cases brief and mainly based on the comparison with a third species, *Oithona spinirostris* Claus, 1863 (= *Oithona plumifera* Baird, 1843). Actually, the first description of the older species *Oithona helgolandica* makes real sense only when simultaneously looking at drawings by the same author of female *Oithona spinirostris* from Messina (Italy) (Claus 1863: Plate XI, figs 4–9). Only the male abdomen, antenna and antennule of *Oithona helgolandica* were figured by Claus in the same plate (Plate XI, figs 10–12). Regarding *Oithona similis*, Claus’s first characterization from specimens collected off Nice was even less complete, and no drawings were provided. Unfortunately, Claus never wrote a comparison of the two species and, to our knowledge, he never deposited any type material for either in a museum.

When studying the copepod fauna from Naples, Giesbrecht (1893) realized that the species *Oithona spinirostris* described by Claus from Messina and Nice, as well as the one that he was himself recording from waters off Naples at that moment, were actually identical to *Oithona plumifera* Baird, 1843, although different from the Kiel specimens he had erroneously identified earlier as *Oithona spinirostris* (Giesbrecht 1881).

Giesbrecht (1893) identified another small form from Naples that he had recorded before as *Oithona similis* Claus off southern Chile and in the Indian Ocean (Giesbrecht 1891a, 1891b). Only then did he notice the close resemblance between the *Oithona similis* specimens from Naples and those collected earlier at Kiel. In fact, only the antennule length prevented him from considering both forms to be identical to *Oithona helgolandica* Claus (Giesbrecht 1893: 539). He described and figured the adult female and male of Neapolitan specimens, thus offering the first detailed description of *Oithona similis* and a comparison with all other congeners recognized at the time. It is worth highlighting that Giesbrecht also commented extensively on the identity and synonymy between *Oithona similis* from Naples and Kiel and *Oithona helgolandica* from Helgoland. Being unable to conclude, he expressed his doubts with question marks when listing synonyms of *Oithona similis* Claus (Giesbrecht 1893 : 537) and suggested the possibility that the majority of the species recorded in the North Atlantic would be (literally) “Oithona helgolandica Claus = ? spinirostris Giesbrecht, 1881 = *Oithona similis* Claus”. As this reads, he gave priority to *Oithona helgolandica* over *Oithona similis*.

Overlooking Giesbrecht’s hesitation and without any factual justification for his judgment, Farran (1913) accepted *Oithona similis* as a good species and excluded *Oithona helgolandica* (and all other synonyms suggested to that date) from his key for the identification of *Oithona* and *Paroithona*. All known species at the time were therein listed and classified based upon the presence/absence of a rostrum and the external exopod setation of the swimming legs. Farran´s deliberate omission of *Oithona helgolandica* and his oversimplification of characters for the classification of *Oithona* species probably have been applied many times until the present day.

In the same year, Sars (1913) recorded the form occurring abundantly in the fjords and offshore waters of Norway as *Oithona helgolandica*. In his opinion, *Oithona helgolandica* was unmistakably identical with *Oithona similis*. Sars stated then that the former name should be retained in accord with the rules of priority. He extended this synonymy to the doubtful species from Kiel that Giesbrecht (1881) had initially identified as *Oithona spinirostris*. It may turn out to be non-trivial that the specimens from New Zealand, also examined by Sars (1913), showed no apparent difference from the northern species. In line with Sars, Scott (1914) also alluded to *Oithona helgolandica* in referring to animals collected off Argentina near to the Malvinas Islands in the SW Atlantic.

In contrast, Rosendorn (1917) named the form he had collected off Chile as *Oithona similis*, although in this case after Giesbrecht’s (1893) description which was based on Neapolitan specimens. While Chilean males fit the description of Mediterranean specimens well, the females differed slightly in the exopod setation of legs 1 and 4. In Rosendorn’s own words, “Giesbrecht probably overlooked the distal outer spine on the third segment of leg 4, as well as one inner seta on the third segment of leg 1” (Rosendorn 1917: 24) (Table 1).

**Table 1.** Worldwide variation in the key characters commonly reported for the determination of *Oithona similis*/*helgolandica* s.l.

Species name^a^ Location Reference	Sex TL	Antennule	Swimming legs setation^b^			Urosome
			P1	P2	P4	
***Oithona helgolandica*** Helgoland (North Sea) (Claus 1863)	F 0.75	“Hardly reaching the end of thorax”	nd	nd	nd	Ur4 shorter than Ur3 and almost as long as Fu. Fu with short setae
***Oithona similis*** Nice (Mediterranean Sea) (Claus 1866)	F ~1.0	“Nearly reaching the base of the urosome”	nd	nd	nd	Fu with short colorless setae
***Oithona spinifrons*** Boeck, 1864 ? *Oithona helgolandica* Claus, 1863 (Brady 1878)	F 0.85	“About as long as the cephalothorax”	nd	nd	nd	Ur1 long, Ur2 and Ur4 about equal and of moderate length, Ur3 somewhat shorter. Fu shorter than any Ur segment
***Oithona similis*** Claus Naples (Mediterranean Sea) (Giesbrecht 1893)	F 0.73–0.80	“Barely to the genital openings”	(0,1,4;1,1,2)/ (1,1,5;0,0,1)	(0,1,5;1,0,1)/ (1,2,5;0,0,1)	(0,1,5;0,0,0)/ (1,2,4;0,0,1)	Ur & Fu relative lengths: 5,12,5,4,5,3.5. CR 2.5 width
	M 0.51–0.61	Geniculate	(0,1,4;1,1,2)/ (nd)	(0,1,4;1,1,2)/ (nd)	(0,1,4;1,1,2)/(nd)	nd
***Oithona similis*** Claus, 1866 Christmas Islands (Indian Ocean) (Farran 1913)	F 0.73–0.80	nd	(nd;1,1,2)/ (nd)	(nd;1,0,1)/ (nd)	(nd)	nd
***Oithona helgolandica*** Claus, 1863 off Norway (Sars 1913–1918)	F 0.70–0.90	“Extending scarcely beyond the anterior division of the body. Length 1.02 times prosome*	(0,1,4;1,1,2)/ (1,6;0,1)	(0,1,5;1,0,1)/ (1,2,5;0,0,1)	(0,1,5;0,0,1?)/ (1,2,4;0,0,1)	Ur & Fu relative lengths: 5,13,5.5,5.5,4.5,4*. CR hardly shorter than Ur4
	M 0.50–0.60	Geniculate	(nd;1,1,2)/(nd)	(0,1,5;1,1,2)/ (1,2,5;0,0,1)	(nd;1,1,2)/(nd)	nd
***Oithona similis*** Claus Valdivia (SE Pacific) (Rosendorn 1917)	F 0.78	“Hardly extending to the genital openings”	(0,1,5;1,1,2)/(nd)	(0,1,5;1,0,1)/ (nd)	(0,1,5;0,0,1)/ (nd)	nd
	M 0.67	Geniculate	(0,1,5;1,1,2)/(nd)	(0,1,5;1,1,2)/(nd)	(0,1,5;1,1,2)/(nd)	nd
***Oithona helgolandica*** Claus [= *Oithona similis* Claus] Adriatic Sea (Pesta 1920)	F 0.73–0.96	“Barely reaching the genital openings”	(nd;1,1,2)/(nd)	(nd;1,0,1)/(nd)	(nd;0,0,0)/(nd)	Fu shorter than Ur4
	M 0.59–0.70	nd	(nd;0,0,2)/(nd)	(nd;0,0,2)/(nd)	(nd;0,0,2)/(nd)	
***Oithona similis*** Claus ?1863 *Oithona helgolandica* Various localities (Kiefer 1929)	F 0.74–0.95	“Reaching the genital openings, located a little before the middle of the genital segment”	(nd;1,1,2)/(nd)	(nd;1,0,1)/(nd)	(nd;0,0,1)/(nd)	nd
	M 0.60–0.70	nd	(nd;1,1,2)/(nd)	(nd;1,1,2)/(nd)	(nd;1,1,2)/(nd)	nd
***Oithona helgolandica*** Claus, 1863 (*Oithona similis* Claus,1863) Various localities (Rose 1933)	F 0.73–0.96	“Barely attains the genital openings”	(nd;1,1,2)/(nd)	(0,1,5;1,0,1)/(nd)	(nd;0,0,0)/(nd)	Fu shorter than Ur4. CR twice width
	M 0.59–0.70	nd	nd	(0,1,5;1,1,2)/(nd)	nd	nd
***Oithona similis*** Claus, 1866 Japan (Mori 1937)	F 0.80	“Reach to the genital pores”	(0,1,4;1,1,2)/(nd)	(0,1,5;1,0,1)/(nd)	(0,1,5;0,0,1)/(nd)	Fu more than twice width. CR equal width
	M 0.65	Geniculate	(0,1,5;1,1,2)/(nd)	(0,1,5;1,1,2)/(nd)	(0,1,5;1,1,2)/(nd)	nd
***Oithona helgolandica*** Claus, 1863 NE Pacific (Davis 1949)	F 0.69–0.96	“Barely reach to genital segment”	(0,1,5;1,1,2)/(nd)	(0,1,5;1,0,1)/(nd)	(0,1,5;0,0,1)/(nd)	Ur 0.75 prosome length. CR shorter than Ur4
	M 0.50–0.70	Geniculate	(0,1,5;1,1,2)/(nd)	(0,1,5;1,1,2)/(nd)	(0,1,5;1,1,2)/(nd)	nd
***Oithona helgolandica*** Claus Messina Strait (Mediterranean Sea) (Crisafi 1959)	F 0.78	nd	(0,1,4;1,1,2)/(nd)	(0,1,5;1,0,1)/(nd)	(0,1,5;0,0,1)/(nd)	nd
	M 0.68	Non geniculate^§^	(0,1,4;1,1,2)/(nd)	(0,1,5;1,0,1)/(nd)	(0,1,5;0,0,1)/(nd)	nd
***Oithona helgolandica*** Claus, 1863 Buenos Aires shelf (Argentine Sea) (Ramírez 1966)	F 0.80	“Reaches the genital openings”	(0,1,4;1,1,2)/(nd)	(0,1,5;1,0,1)/(nd)	(0,1,5;0,0,1)/(nd)	Ur1 2.5 width. Ur4 similar to Ur2 and Ur3. Fu slightly shorter than Ur4. CR twice width
***Oithona similis*** Claus, 1866 Río Deseado estuary (Argentine Sea) (Pallares 1968)	F 0.89–1.10	nd	(nd;1,1,2)/(nd)	(nd;1,0,1)/(nd)	(nd;0,0,1)/(nd)	CR divergent
	M 0.50–0.67	Geniculate	nd	nd	nd	nd
***Oithona helgolandica*** *sensu* Sars, 1913 Gulf of Lion (Mediterranean Sea) (Razouls 1972)	F nd	nd	nd	(0,1,5;1,0,1)/ (1,2,5;0,0,1)	(0,1,5;0,0,0)/ (1,2,4;0,0,1)	nd
	M nd	nd	(0,1,3;1,1,2)/ (1,1,5;0,0,1)	(0,1,5;1,1,2)/ (1,2,5;0,0,1)	(0,1,5;1,1,2)/ (1,2,4;0,0,1)	nd
***Oithona similis*** Claus, 1866 Suruga Bay (Japan) (Nishida et al. 1977)	F 0.69–0.84	“Extending to the end of thorax 5”	(0,1,4;1,1,2)/ (1,1,5;0,0,1)	(1,0,5;1,0,1)/ (1,2,5;0,0,1)	(0,1,5;0,0,1)/ (1,2,4;0,0,1)	nd
	M 0.60–0.65	nd	(0,1,4/5;1,1,2)/ (nd)	(0,1,5;1,1,2)/(nd)	(0,1,5;1,1,2)/(nd)	nd
***Oithona similis*** Claus, 1866 ? *Oithona helgolandica* Claus, 1863 Various localities (Shuvalov 1980)	F 0.78	nd	(0,1,4;1,1,2)/ (1,1,5;0,0,1)	(0,1,5;1,0,1)/(nd)	(nd;0,0,1)/(nd)	nd
	M 0.60–0.70	nd	nd	nd	nd	nd
***Oithona similis*** Claus, 1866 (SW Atlantic) (Björnberg 1981)	F nd	Extending slightly beyond thorax 5*	nd	nd	nd	nd
	M 0.70	nd	(nd;1,1,2)/(nd)	(nd;1,1,2)/(nd)	(nd;1,1,2)/(nd)	nd
***Oithona similis*** Claus, 1866 Various localities (Nishida 1985)	F 0.68–0.96	“Length 1.1–1.3 times prosome”	(0,1,4;1,1,2)/ (1,1,5;0,0,1)	(0,1,5;1,0,1)/ (0/1,1/2,5;0,0,1)	(0,1,5;0,0,1)/ (1,2,4;0,0,1)	Ur & Fu relative lengths: 13,34,15,14,14,11. Ur4 1.1–1.3 width. CR 1.9–2.4 width
***Oithona similis*** Claus, 1866 Magallanes Strait (Argentina-Chile) (Mazzochi et al. 1995)	F 0.80–0.92	nd	(0,1,4;1,1,2)/ (1,1,5;0,0,1)	(0,1,5;1,0,1)/ (1,2,5;0,0,1)	(0,1,5;0,0,1)/ (1,2,4;0,0,1)	Ur & Fu relative lengths: 15,36,14,12,12,11
**Oithona aff. helgolandica** *sensu* Sars, 1913 Buenos Aires and southern Patagonian shelves (Argentine Sea) (Our unpublished data)	F	Extending to the genital openings. Length 1.1-times prosome.	(0,1,4;1,1,2)/ (1,6;0,1)	(0,1,5;1,0,1)/ (1,2,5;0,0,1)	(0,1,5;0,0,1)/ (1,2,4;0,0,1)	Ur & Fu relative lengths: 13.5,34,16,14,13,10.5. Ur1 2.0–2.2 width. CR twice width
	M	Geniculate	(0,1,4;1,1,2)/nd	(0,1,5;1,1,2)/nd	(0,1,5;1,1,2)/nd	nd

In a surprising twist, Sars (1918) radically changed his former opinion and, “on a closer consideration,” he concluded that “the two forms recorded by Claus under the names *Oithona helgolandica* and *Oithona similis* are in reality very distinct species, the former being in all probability identical with the form subsequently described by Giesbrecht as *Oithona nana*, which accordingly must bear the older name *helgolandica*.” For the Norwegian form “the specific name *similis* given by Claus ought of course to be retained” (Sars 1918: 207).

More recently, Crisafi (1959) reviewed the historical sequence and concluded that *Oithona similis* should be regarded as synonymous with *Oithona helgolandica* on the grounds that the singular characters for the junior name, *Oithona similis*, in Claus’s diagnosis were insufficient to establish a new species. Nishida et al. (1977: 151) also discussed the issue but suggested, on the contrary, that the name *helgolandica* “should be rejected because of uncertainty and that Giesbrecht’s (1893) description of *Oithona similis* is accepted as a good species”. As did Crisafi (1959), we believe that Claus was unable to find the set of differential characters that would have been necessary for the proposal of a new species. He probably described under the new name *similis* individuals that were similar, though not identical, to the species he found formerly in Helgoland.

Given this state of the problem, many authors have subsequently either applied the Principle of Priority or followed Crisafi’s (1959) opinion, naming the species *Oithona helgolandica* (e.g., Pesta 1920; Rose 1933; Davis 1949; Crisafi 1959; Ramírez 1966, 1970; Razouls 1972; Huys and Boxshall 1991). Many others have preferred to refer to *Oithona similis* (e.g., Farran 1929; Kiefer 1929; Mori 1937; Rose 1957; Nishida et al. 1977; Shuvalov 1980; Nishida 1985), although some of those indicated with question marks their doubts about synonymy with the senior form *Oithona helgolandica*. This ambiguity has continued until the present day.

## Do the names *Oithona similis* and *Oithona helgolandica* refer to identical taxa?

Most important morphological features usually used for the identification of *Oithona similis* / *helgolandica* s.l. have been: (i) body size, (ii) rostrum presence and direction, (iii) relative antennule length, (iv) exopod setation of swimming legs 1-4, and (v) relative lengths of the genital segment, anal segment, and furcae.

**Figure 1.**
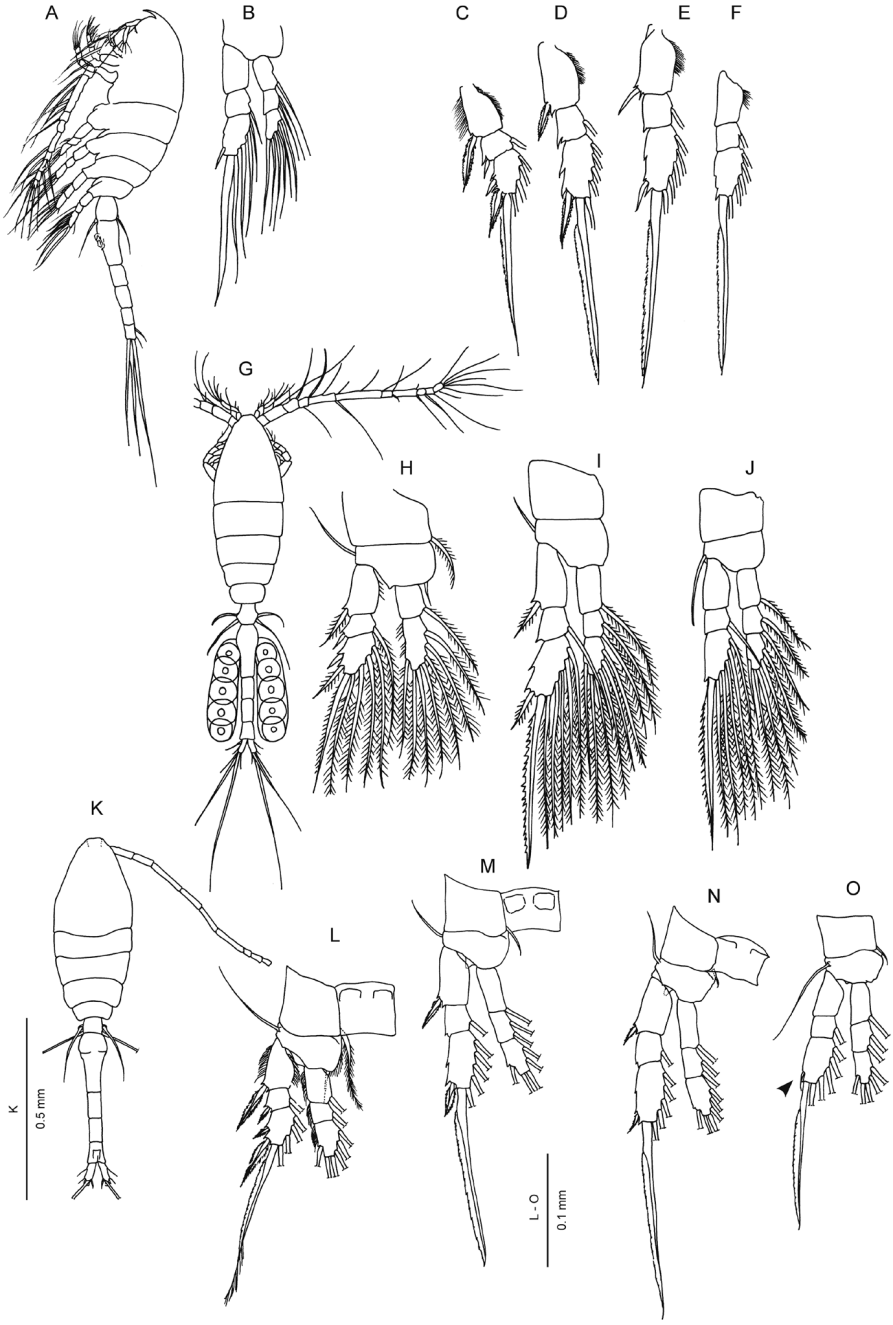
Former selected drawings of *Oithona similis* / *helgolandica*. **A, B** *Oithona spinifrons* Boeck, 1864 (=? *Oithona helgolandica* Claus), female body and “one of swimming feet” (= leg 4?) (after Brady1878, Plates XIV and XXIV A) **C–F** *Oithona similis* exopod of legs 1 to 4 (after Gisbrecht 1893, Plate 34) **G–J** *Oithona helgolandica*, female body, legs 1-2 and leg 4 (after Sars 1913, Plate III) **K**–**O** *Oithona similis*, female body and legs 1 to 4 (after Nishida 1985, fig. 50 and 51). Original illustrations were faithfully copied in all details and rearranged to facilitate comparisons. Scale bars only provided in Nishida (1985).

Morphological differences among specimens worldwide (Table 1; Fig. 1) suggest that at least two forms may be referred to *Oithona similis* / *helgolandica* s.l. Strict comparisons across records are not really possible, because they all lack the detail of one or more particular key characters; hence, it seems likely that identification of *Oithona similis* s.l. / *helgolandica* s.l. has generally been based on elements insufficient for adequate taxonomic judgment. This is not minor when considering that phenetically similar species may differ from one another in only slight differences of the setal formula of the swimming legs (Nishida 1985). We are calling attention here to the fact that the female and male exopod setation of swimming legs do not match identically in the two most complete and detailed redescriptions of *Oithona similis* by Giesbrecht (1893) and Nishida (1985), and neither is there complete correlation between Nishida et al (1977) and Nishida (1985) (Table 1).

Some subtle differences are apparent among published drawings labelled as *Oithona similis* s.l. and of *Oithona helgolandica* s.l. (Fig. 1). In this regard, it may be worth examining closely the drawings of female *Oithona helgolandica* by Sars (1913, Plate III) and *Oithona spinifrons* Boeck, 1864 (= ? *Oithona helgolandica* Claus) by Brady (1878, Plates XIV and XXIV A). In considering *Oithona similis* as figured by Nishida (1985), note in particular the dissimilar general appearance with respect to the above mentioned species, the two-segmented endopod of the first leg, and the overall setation of legs 1–4 (on the inner and outer borders of both rami). Nishida’s descriptions and drawings probably correspond to the “typical” *Oithona similis*, on which a substantial number of authors have based identifications since 1985.

In our view, when specimens have been identified as *Oithona similis* s.l., insufficient attention has often been paid to: (i) presence/absence of the small distal outer spine on exopod segment 3 of leg 4, (ii) endopod segmentation of leg 1, and (iii) relative antennule length.

The distal outer spine on the last segment of the exopod of leg 4 is lacking in some early descriptions and drawings of *Oithona similis* / *helgolandica* (e.g., Giesbrecht 1893; Pesta 1920; Rose 1933). Sars (1913) gives account of it in the text (p. 8) but it is unclear in his drawing. According to Farran (1913) and Crisafi (1959), this spine can be easily lost, although it may also have been overlooked, as Rosendorn (1917) suggested. From records in the literature, doubt remains whether or not all Mediterranean specimens share this particular character, the presence of this spine (Table 1).

In the genus *Oithona* Baird, both rami of the first swimming leg are 3-segmented (*sensu* Brady 1878), but this is not always the case in *Oithona similis* s.l. and *Oithona helgolandica* s.l. To our knowledge, a bi-segmented endopod of leg 1 has only been specifically reported so far for female *Oithona* specimens from Norway (Sars 1913), the Gulf of Lion (Razouls 1972), and off Argentina (our unpublished data). Like most authors listed in Table 1, Giesbrecht (1893) only figured the outer rami of the swimming legs, though he addressed secondarily his observation that “segments 2 and 3 of the endopod were often indistinctly separated in the front pairs of *Oithona similis*” (on p. 544).

Lastly, the antennule length relative to the prosome appears slightly variable across records worldwide (Table 1). Because this feature has been most often characterized in a subjective way, it is suggested that it be reported quantitatively in the future (e.g., antennule length 1.1–1.3 times prosome length, as reported by Nishida 1985).

From the genetic point of view, the still scarce molecular studies on *Oithona* also support the hypothesis that more than one form is reported under the same specific name, *Oithona similis* s.l. Cepeda et al. (2012) presented data showing significant genetic differentiation among numerous and widespread locations in the North and South Atlantic Ocean based on 28S rDNA, suggesting some degree of isolation amongst sampled populations. Furthermore, preliminary findings from cytochrome c oxidase I (COI) “barcode” analyses of (apparently) morphologically identical *Oithona similis* over a broad geographical scale, i.e. Arctic and Southern oceans, North Sea and Mediterranean Sea, revealed the presence of several different haplotypes restricted to particular areas (Wend-Heckmann 2013). There is thus the possibility that *Oithona similis* s.l. is not a single, broadly distributed, cosmopolitan species but rather, a conglomerate of several cryptic species. This has been the case of many putatively cosmopolitan species (Bucklin et al. 2011). In this regard, markers frequently used to investigate boundary taxa among closely related, cryptic and cosmopolitan species may be helpful (e.g., COI, cytochrome b, 16S rRNA, Internal Transcribed Spacer 1–2).

## Nomenclatural remarks and perspectives

The nomenclatural implications of the taxonomic uncertainty apparent from the discussions above are not minor. From a historical standpoint, it is clear that over the course of time a substantial number of copepodologists has come to consider that *Oithona similis* and *Oithona helgolandica* actually denote the same taxon. Prevailing use which, as shown, has depended upon individual judgment and opinion, has made that the junior synonym *Oithona similis* were very commonly imposed over the older *Oithona helgolandica*, contradicting the rules of priority (ICZN 1999; Article 23).

On the other hand, morphological differences worldwide in the key characters commonly used for diagnosis (Table 1) suggest that *Oithona similis* and *Oithona helgolandica* may not refer to copepods related closely enough to be considered a single taxon. Because the problem focuses on the identity of Claus’s types, which unfortunately are not available, we advocate a thorough comparison of the two taxonomic entities, preferably at both the morphological and genetic levels (McManus and Katz 2009), from specimens newly collected at their respective type localities, i.e, Nice and Helgoland.

In the absence of proper holotypes, the designation of neotypes probably will be required because of the points raised above (ICZN 1999; Article. 75), i.e.:

(i) A neotype each for *Oithona helgolandica* s.s. Claus, 1863 and for *Oithona similis* s.s. Claus, 1866 will be needed if specimens from both localities are proved to be different.

(ii) The appointment of only one neotype will be necessary if specimens from Nice and Helgoland are substantially identical. Strictly speaking, in this situation the senior name *Oithona helgolandica* should be used because of the rules of priority. Nevertheless, in pursuit of stability and universality and to avoid causing further confusion, it would be still possible to maintain the use of the junior synonym, *Oithona similis*, as it has largely prevailed through time. To stabilise this, however, the matter must be referred to the ICZN for a ruling under the plenary powers (ICZN 1999; Article 23.9.3).

There are not, in fact, conclusive fundamentals at present in support of an objective synonymy between the names *Oithona similis* and *Oithona helgolandica*. Hence, until the diagnostic characters are re-examined and the nomenclatural issues settled, we strongly recommend specifically reporting the authority upon which the identification of either *Oithona similis* s.l. or *Oithona helgolandica* s.l. has been undertaken. In this process, particular reference should be made for female specimens in respect to: (i) relative antennule length, (ii) presence/absence of the small distal outer spine on exopod segment 3 of leg 4, and (iii) endopod segmentation of leg 1.

After this review, we find astounding the extent of taxonomic and nomenclatural uncertainty surrounding the name *Oithona similis*. Poor original diagnosis and frequently the inability of authors to perceive minute morphological differences have very likely caused the assembly of several forms distinct at the species level into a single, nominal species. This circumstance on top of the persistent confusion with its likely sibling, *Oithona helgolandica*, may have led to a false impression of cosmopolitanism. It is possible that many cryptic species are veiled behind the apparent morphological homogeneity of their forms, and *Oithona similis* s.l. and *Oithona helgolandica* s.l. may be an example in an abundant and ecologically important group, the genus *Oithona*. Therefore, we encourage a profound revision of *Oithona similis* s.l. in order to bring the exact status of this species to light. In accomplishing this goal, species should not be renamed or newly assigned based on morphology alone without the support of molecular genetic sequence information.
