# Corrigenda: Cepeda GD, Sabatini ME, Scioscia CL, Ramírez FC, Viñas MD (2016) On the uncertainty beneath the name *Oithona similis* Claus, 1866 (Copepoda, Cyclopoida). ZooKeys 552: 1–15. doi: 10.3897/zookeys.552.6083

**DOI:** 10.3897/zookeys.565.8009

**Published:** 2016-02-17

**Authors:** Georgina D. Cepeda, Marina E. Sabatini, Cristina L. Scioscia, Fernando C. Ramírez, María D. Viñas

**Affiliations:** 1 Instituto de Investigaciones Marinas y Costeras (IIMyC), Facultad de Ciencias Exactas y Naturales, Universidad Nacional de Mar del Plata, Consejo Nacional de Investigaciones Científicas y Técnicas (CONICET), Funes 3350, B7602AYL Mar del Plata, Argentina; 2 Instituto Nacional de Investigación y Desarrollo Pesquero (INIDEP), Paseo Victoria Ocampo No 1, B7602HSA Mar del Plata, Argentina; 3 Museo Argentino de Ciencias Naturales Bernardino Rivadavia (CONICET-MACNBR), Avenida Ángel Gallardo 470, C1405DJR Buenos Aires, Argentina

It came to our attention after our manuscript was published that the caption of Table 1 was incomplete. We provide below the missing information, which is essential to the correct interpretation of the referred table.

^a^ Species names and authors are as specified in the original text.

^b^ Setation formulae of the first (P1), second (P2) and fourth (P4) swimming legs are summarized as follows: Re (inner setae; outer setae)/Ri (inner setae; outer setae), where Re: exopod, Ri: endopod. F: adult female; M: adult male; TL: total length (mm); Ur1 to Ur5: urosome segments; Fu: furca; CR: caudal rami. nd: no data.

* Character not explicitly stated in the original but taken from accompanying drawings for comparison purposes.

^§^ Most likely Crisafi (1959) described a late juvenile C5 as an adult male. In addition to the non-geniculated antennule, the urosome is 4-segmented with the last two segments fused (Fig. 3, p. 51 in Crisafi, 1959).

